# Attitudes towards sickness absence and sickness presenteeism in health and care sectors in Norway and Denmark: a qualitative study

**DOI:** 10.1186/1471-2458-14-880

**Published:** 2014-08-27

**Authors:** Line Krane, Eva Ladekjær Larsen, Claus Vinther Nielsen, Christina Malmose Stapelfeldt, Roar Johnsen, Mette Bech Risør

**Affiliations:** Department of Community Medicine, UiT The Arctic University of Norway, 9037 Tromsø, Norway; Marselisborg Centret/Public Health and Quality improvement, Central Denmark Region, Aarhus, Denmark; Unit for Health Promotion Research, University of Southern Denmark, Esbjerg, Denmark; Section of Social Medicine and Rehabilitation, Department of Public Health, Aarhus University, Aarhus, Denmark; Department of Public health and General Practice, University of Trondheim, Trondheim, Norway

**Keywords:** Sickness absence, Sickness presenteeism, Attitudes, Moral aspects, Concept of work, Focus group discussions

## Abstract

**Background:**

In the health and care sector, sickness absence and sickness presenteeism are frequent phenomena and constitute a field in need of exploration. Attitudes towards sickness absence involve also attitudes towards sickness presenteeism, i.e. going to work while sick, confirmed by previous studies. Sickness behavior, reflecting attitudes on work absence, could differ between countries and influence absence rates. But little is known about attitudes towards sickness absence and sickness presenteeism in the health and care sectors in Norway and Denmark. The aim of the present paper is therefore to explore attitudes towards sickness absence and sickness presenteeism among nursing home employees in both countries.

**Methods:**

Eight focus group discussions (FGDs) were conducted using a semi-structured interview guide, the main attention of which was attitudes towards sickness absence and sickness presenteeism. FGDs were conducted in two nursing homes in Norway and two in Denmark, with different geographic locations: one in a rural area and one in an urban area in each country. FGDs were recorded, transcribed and analyzed using framework analysis to identify major themes and explanatory patterns.

**Results:**

Four major significant themes were identified from the FGDs: a) sickness absence and sickness presenteeism, b) acceptable causes of sickness absence, c) job identity, and d) organization of work and physical aspects of the workplace. Our analyses showed that social commitment and loyalty to residents and colleagues was important for sickness absence and sickness presenteeism, as were perceived acceptable and non-acceptable reasons for sickness absence. Organization of work and physical aspects of the workplace were also found to have an influence on attitudes towards sickness absence.

**Conclusions:**

The general interpretation of the findings was that attitudes towards sickness absence and sickness presenteeism among nursing home employees were embedded in situational patterns of moral relationships and were connected to a specific job identity. These patterns were constituted by the perception of colleagues, the social commitment to residents, and they influence on what was deemed as acceptable and non-acceptable reasons for sickness absence. In other words, attitudes towards sickness absence and sickness presenteeism were socially and morally determined at personal levels by an overall concept of work, independent of country.

## Background

Sickness absence is an extensive phenomenon determined by several factors, including employee health, the health care system, work environment and individual factors [1], and is of considerable concern in many Western countries [2]. Structural aspects like legislative and economic frameworks can greatly influence sickness absence rates [2, 3], and job security has also proven to be important. The causes of sickness absence are many and complex [4]. However, the important relational and moral aspects of illness behavior have been only sparsely studied [5].

Sickness presenteeism, as the opposite phenomenon to sickness absence, can be defined as going to work despite judging that one’s state of health is poor enough to justify sick leave [6, 7], and has usually been considered a complementary alternative to sickness absence [8, 9]. In a random sample of the Swedish work force, 44% of workers in the health and care sectors reported sickness presenteeism on more than one occasion during the preceding year [7]. Although sickness presenteeism rates differ from sector to sector, employees whose job tasks included caring for or interacting with people were reported to be more likely to go to work while sick than employees with other occupations [10]. The health and care, and educational sectors have the highest sickness presenteeism, and the association between difficulties in finding replacements or temporary workers and presenteeism has been confirmed in different studies [6, 7, 11]. Repeated sickness presenteeism is associated with subsequent long-term sickness absence [12], and sickness presenteeism is an independent risk factor for fair/poor future general health [13]. Gustafsson et al. [14] suggested that both sickness absence and sickness presenteeism are strong predictors of poor future health, physical complaints, low mental well-being and low work ability.

Previous studies have shown an association between sickness absence and sickness presenteeism [15, 16]. This is supported by the findings of Leineweber [8], who reported a correlation between sickness absence and sickness presenteeism.

Hansen [15] showed that employees who took sickness absence were more likely to have been in a situation where they went to work despite believing they could legitimately have stayed at home. Both sickness absence and sickness presenteeism seem to be related to various aspects of one’s work situation [17], and associations between sickness presenteeism and high workload, time pressure and job insecurity have been observed in Nordic elderly care [18].

The relationship between the concepts of illness, disease and sickness can be studied to better understand how sickness absence and sickness presenteeism are inter-related, as these concepts are used to capture different aspects of ill health [19, 20]. An exploration by Wikman [19] showed a low degree of overlap between illness, disease and sickness, and sickness absence over time, indicating that they represent different realities and different aspects of morbidity. The ability to work despite an illness or disease also depends on the type of work and work demands, therefore sickness absence not only reveals certain aspects of an illness, but also whether one’s work environment can be adapted, and one’s work load adequately compensated, in times of sickness absence [7, 15, 19]. For example, work in the health and care sectors consists largely of caring for other people, and of specific tasks that often cannot be postponed. There are few possibilities to choose work tasks, or to work at a slower pace, which makes sickness presenteeism among these employees difficult. Nevertheless, illness is not necessarily followed by sickness absence in the health and care sectors; instead sickness absence and sickness presenteeism are alternative illness behaviors [17].

Moral aspects of sickness absence and the social control this can engender in the workplace have been scarcely studied [5]. For example, perceived acceptable and non-acceptable causes of sickness absence often reflect an underlying moral evaluation by colleagues and superiors as to what degree of illness merits sickness absence. This moral evaluation of illness behavior can lead to the attribution of at least a moral position, if not an explicit label [5]. A moral position can be described as a characteristic attributed to a colleague by another colleague or superior. These attributions may develop into a strategy of social control, whereby a colleague’s illness becomes a pretext to justify additional judgments related to other aspects of his or her life in the workplace. Overall, such deliberations spring from other traditions than the epidemiological studies mostly mentioned here. Here qualitative analysis based on interview data and a grounded theory approach was used by Dodier [5].

Epidemiological studies e.g. show that sickness absence rates are higher in Norway compared to Denmark, respectively 7.1% and 5.2% [21–23], and in both countries sickness absence rates are among the highest in the health and care sectors [24, 25]. One study found that there were more nurses employed in the health and care sector in Norway compared to Denmark, however there were more nursing assistants employed in Denmark [26]. The sickness absence rates in the health and care sectors were 11.3% in Norway and 7.0% in Denmark. In both countries the large majority of employees in this sector were women [26]. Because of higher sickness absence rates in Norway, it has been hypothesized that Norwegian health and care sector employees have different attitudes towards sickness absence and sickness presenteeism compared to Danish health and care sector employees. In addition, there is a high proportion of sickness absence among younger employees in both countries, and a higher proportion of sickness absence among older employees in Norway compared to Denmark, indicating differences in attitudes according to age. One study showed a clear tendency for younger individuals to be more liberal towards sickness absence than older persons [27]. Disclosing attitudes towards sickness absence and presenteeism is of societal interest, as they will influence the effects of regulations of sick leave legislation.

The aim of this study was to explore attitudes towards sickness absence and sickness presenteeism by interviewing nursing home employees in varying social and structural settings. In order to gain more insight into the dilemmas faced by these employees as a result of sickness absence and sickness presenteeism, a qualitative approach was chosen, focusing on the pertaining attitudes and experiences of the participants.

## Methods

Information was collected from focus group discussions (FGDs) carried out in Norway and Denmark using a semi-structured interview guide, the main attention of which was attitudes towards sickness absence and sickness presenteeism. Norway and Denmark was chosen due to differences in sickness absence and sickness presenteeism as explained in the Background, but they do not constitute the basis for a rigorous comparison between countries. Instead the role of the countries is to supplement the analysis with contextual information (political regulations, workplace management, employment conditions, geographic location etc.) as reflections on the results.

FGDs are useful when exploring differences in opinion, and allow for the elaboration of a given phenomenon through interactive discussion of participants’ experiences and attitudes in a group setting [28]. Because the purpose of this study was to gain insight into employees’ attitudes, perceptions and thoughts about sickness absence [28–30], and to encourage them to discuss and explain how they handled sickness absence and sickness presenteeism in a meaningful way between colleagues, the FGD approach was used. Furthermore, through the FGDs, we wanted to see if there were differences in attitudes among employees, e.g. according to age, reasons for absenteeism, and perception of the management level.

### Recruitment procedure and material

Invitation letters were sent to one municipality in each country, which was responsible for selecting the nursing homes that would be included in the study. The administration of the nursing homes then selected the focus group participants according to information from the researchers on how the groups should be composed. They were instructed to divide the groups into a younger group less than 40 years old, plus an elderly group more than 50 years old to get the groups most different from each other, and the groups should preferably consist of four to six nursing assistants. Both sexes could be represented. Largely, we got a range of participants of different age, different seniority, different work experience and a slight difference in training. As such, the management provided a mix of participants representing many different attitudes.

Eight semi-structured FGDs were conducted in four nursing homes: two in Norway and two in Denmark. Two FGDs were conducted in each nursing home, and the four nursing homes had different geographic locations, with one in rural area and one in an urban area in both Norway and Denmark. A team of two researchers, one Norwegian and one Danish, led all FGD’s. The Norwegian researcher was the moderator in the FGDs in Norway, and the Danish researcher was the assistant moderator, and their roles were reversed in the FGDs in Denmark. The interviews were performed in Norwegian and Danish according to country.

The division into age groups did not turn out the way we expected for some of the FGD (Table 1). Further, focus groups consisted of two to six participants (Table 1). The educational level in this study varied from nursing assistants (1–2 years at the secondary level with internship) to nurses (3 years at the college level). However, nursing assistants were the most prevalent in our sample. Only two nurses participated in two of the FGDs (one nurse in each interview). The work tasks of both nursing assistants and nurses consisted of caring for the residents of the nursing homes; none had supervisory responsibilities.

**Table 1:** **The eight focus groups which constitute the data material**

	Norway				Denmark			
	Rural area		Urban area		Rural area		Urban area	
**Age of participants**	Group A	Group B	Group C	Group D	Group A	Group B	Group C	Group D
	40–48 years old^1^	52–65 years old	21–27 years old	58–66 years old	In their 30s	In their 40 s^2^	In their 20s	23–43 years old
**Work tasks**	Caring for residents in nursing homes	Caring for residents in nursing homes	Caring for residents in nursing homes	Caring for residents in nursing homes	Caring for residents in nursing homes	Caring for residents in nursing homes	Caring for residents in nursing homes	Caring for residents in nursing homes
**Qualifications**	Nursing assistants, nurse	Nursing assistants	Nursing assistants	Nursing assistants	Nursing assistants	Nursing assistants	Nursing assistants	Nursing assistants
**Group number**	1 a, NO	1 b, NO	2 a, NO	2 b, NO	3 a, DK	3 b, DK	4 a, DK	4 b, DK

^1^Few younger than 40 years old at this nursing home.

^2^Few older than 50 years old at this nursing home.

The age of the participants ranged from 21 to 66 years old, and all participants were women, due to the fact that very few men work in nursing homes in the two countries.

The semi-structured interview guide covered the following topics: description of a typical work day, management of sickness absence, causes of sickness absence, sickness presenteeism, length of sickness absence (long or short as per the employee perception), management and perception of sickness absence by colleagues (practically, morally and socially), job acknowledgement by superiors and colleagues and job satisfaction. Each interview lasted between 1 and 1-1/2 hours. We interviewed the participants during the work day at the nursing home for all FGDs. Due to sickness absence, one of the FGDs was completed with only two participants.

### Analysis

All interviews were recorded, transcribed and analyzed using Framework Analysis to identify major themes, explanatory patterns and perspectives on sickness absence [31, 32]. Framework Analysis is a systematic process of sifting, charting and sorting material according to key issues and themes, and its process of analysis and interpretation ensures transparency in the analytical process [31]. Moreover, Framework Analysis was developed for applied policy research, e.g. research that meets specific information needs and may inform future actions or interventions [31]. We found this method useful for exploring and interpreting our data, as our study is part of a larger study aiming to inform policy [26, 31, 32]. As mentioned, the analysis did not aim for a rigorous comparison between countries, but saw the FGDs as a whole as variations over the research aim.

To explore and validate coding and inferences and to substantiate the interpretation of data in this study, we planned and developed a workshop of three days with selected authors (MBR, ELL and LK). MBR had not been part of the interview design before the study took place, but assisted in coding. ELL had performed FGDs in Denmark but had not been part of the coding process until the workshop. As such, all represented different critical positions towards validity. The aim of the workshop was to discuss and agree on the framework, index, and the interpretation of the perspectives. An initial coding framework was developed from the topics in the semi-structured interview guide using the Framework Analysis approach. To identify the main themes we used familiarization, i.e. got to know the material, developed a thematic framework (Table 2), and then used an indexing process when transferring data into the thematic framework. Data was lifted from its original context and a chart containing the different dimensions of the index items was developed to create a picture of the data. The last step was to interpret the perspectives as a whole, i.e. their explanatory content and the relationships between emerging themes, i.e. the specified attitudes and reflections on sickness absence. Sections of the transcribed interviews are cited in italics. The symbol ‘…’ represents hesitation on the part of the speaker, ‘…[…]…’ represents omitted text, and text in square brackets ‘[]’ represent author’s notes. The data program NVivo 9.2 was used to organize, code and index the qualitative data.

**Table 2:** **Themes identified in the interviews and used in the framework analysis**

Theme	Topic/description
**Sickness absence and presenteeism**	
	Sick colleague
	Social problems
	Collective normativity of sick leave
	High/low sickness absence
	How ill should one be in order to report sick?
	Age and sickness absence
	Rearrangement by the use of temporary staff
	What happens practically when an employee report sick: sick calls, sickness presenteeism, use of temporary staff
**Acceptable causes of sickness absence**	
	Does one know why a colleague is sick?
	Acceptable reasons for sick leave
	Non-acceptable causes of sickness absence
	Stress
	Strain injury
	Infection
**Job identity**	
	Relation to resident
	Relation to colleague
	Relation to management
	Job satisfaction
	Influence on job
	Good working day
**Organization of work and physical aspects of the workplace**	
	Start of the working day
	Changing tasks and groups
	Facilitation
	Age composition
	Percent of employment
	Education
	Freeze of staff recruitment
	Shifts
	Bullying
	Length of employment
	Work environment
	Recognition from management
	Urban/rural area
	Size of nursing home
	Old building
	Several floors
	Lunch room facility

### Ethics and consent

The selected health care employees received an invitation letter, informing them of the aim and confidentiality of the study. Informed consent was obtained before FGDs were initiated. The project was approved by the Data Protection Official for Research through the Norwegian Social Science Data Service (approval number 24090). The project was subject to the rules for processing personal data, see § 7–27 of the Personal Data Regulations. Approval (2012-41-1290) for conducting this study was also given by the Danish Data Protecting Agency.

## Results

The analysis of the FGDs resulted in four major themes with several topics/descriptions (Table 2), which covered the topics spontaneously discussed by the FGD participants, as well as discussions related to direct questions posed from the semi-structured interview guide. The four most significant themes that emerged from the FGDs were: sickness absence and sickness presenteeism, acceptable causes of sickness absence, job identity, organization of work and physical aspects of the workplace (i.e., age of the building, layout, number of floors, etc.). The age distribution in the focus groups turned out to be different than intended, so only when issues obviously are related to age, it is mentioned. All the names mentioned in the study are fictitious to protect the identity of the participants.

### Sickness absence and sickness presenteeism

Sickness absence and sickness presenteeism were discussed and commented upon *simultaneously* by focus group participants as two sides of the same issue. There was a mix of topics expressed, with sickness as the overall theme. Participants gave their personal and moral opinions about who was sick, what the colleague could and could not do at work, the cause of the sickness absence and the implications of staying at home and of going to work. These opinions were based on the relationships between employees, which were important in relation to sickness absence and sickness presenteeism.

When employees went to work sick at any of the nursing homes, there was little chance of being spared heavy tasks because there was much to do, and only seldom did they have a medical certificate or notice from their supervisor saying some tasks were not required of them. Indeed, the participants gave several examples of situations where they themselves, or their colleagues, went to work while sick and were not able to do their work properly. Overall, it was seen as difficult to have colleagues who were sick at work, because everyone was already experiencing high strain and they lacked the energy to take on their colleagues’ work tasks in addition to their own.

*…I’ve never just said to colleagues that I’ll only be able to do certain things. I’ve seen a doctor first and then talked to Anne, and then she’s written - it’s always like that with pregnant women as well - "They must do this and that" or "They can’t, …[…]… but I don’t think, because of the workload, that you’ll then say "Sweetheart, come here…", I don’t think so. But then again, that’s also because you’re wound a little tight, and then it’s kind of…well, there’s not much to give. I think you try to be nice to them, but if you’re feeling like crap and coming in, it’s not okay…*

*Rural nursing home, group A, Denmark*

The work load was reported to be high, such that the employees were not able to take on extra tasks when colleagues came to work sick, even if they wanted to. All focus groups discussed this and expressed that the employees were already at the limit of their workload capacity.

In situations where it was not possible to get temporary employees to replace them, participants questioned if it was better to go to work while sick, or to stay at home. One of the participants described how the moral evaluations were done:

*…we were really stressed out and had loads to do, and then there were people who were not supposed to do much, because their job description said something else [*was temporarily changed*], and there were tasks that they should not do. And that really sours the mood, because you can see them sitting down, enjoying the company of the residents, doing something else, and the rest of us are just working our fingers to the bone …[…]… So, I think that regardless of how much you understand that someone might not be feeling great and all that, at some point you just snap.*

*Rural nursing home, group B, Denmark*

Our data showed that the presence of a partly sick colleague at work without extra temporary help might work out for a while, but over time the extra burden could be too much for the healthy colleagues and the work environment might suffer. One participant said that it was not reasonable to expect her to do the hard work while a partly sick colleague did only the easy tasks. The participant expressed the moral expectation that if one came to work, one should be able to perform the work tasks required, and if that was not the case one should stay at home.

*… When I’m getting four up [*help the residents getting up from bed, get dressed and go to breakfast*], and then I’ll have to get the other four up, and she just has to make sandwiches? That’s not right… So, I feel that if you’re at work, you have to be able to work, if not, you have no business being there.*

*Urban nursing home, group D, Norway*

Participants in all FGDs expressed similar attitudes towards going to work while partly sick. They claimed that it was difficult to be partly sick at work because of the type of work they did; that as they had to serve their residents and had tasks that were impossible to postpone, one had to be able to work 100 percent, or your colleagues would suffer a heavier workload. The participants expressed this in terms of a strong commitment to their residents and their colleagues, saying that if you went to work partly sick, you would easily have a bad conscience towards your colleagues. One participant expressed it like this:

*… So, our policy is that you can [*come to work when you’re sick*], and then just say "I can’t do this today" …[…]… But then, you feel guilty if you say, if you say to your colleagues "I can’t…"*

*Urban nursing home, group D, Denmark*

When going to work while partly sick, many participants said they found themselves in a dilemma with regard to their healthy colleagues – they wanted to be loyal and helpful, but at the same time had a bad conscience for showing up sick, both towards themselves and for fear of contaminating others. However, they also had bad conscience if they didn’t go to work, because they knew their colleagues would have a heavier workload. This dilemma born of commitment and moral responsibility to residents and colleagues was often reported by our participants. One participant expressed the time pressure and commitment to the residents like this:

*Instead of thinking about the person you’re supposed to take care of, they almost come last, and that puts pressure on you mentally. When you go to bed at night, you think "Did I do something for this person today, or that person?" Right? And, how do the residents perceive me? Like a storm that blows in, and then leaves again?*

*Urban nursing home, group D, Norway*

Closely related to the above was how the nursing homes’ administrations managed sickness absence; whether temporary employees were summoned to replace those who called in sick, and if so for how long. Normally, employees called in sick as early as possible in the morning, but only occasionally was a temporary worker called in. The use of temporary workers seemed to differ across nursing homes. Employees in nursing homes in Denmark described their perception that the budget was tighter than in Norway, and had an idea that it was more common to summon temporary workers in Norway than in Denmark.

Our participants to a limited extent cited the age aspect of sickness absence as a theme. When participants were asked by the moderator who they thought had most sickness absence, many participants replied it was the temporary workers; the age aspect of sickness absence was not seen as a main concern.

### Acceptable causes of sickness absence

Causes of sickness absence were discussed and defined as being acceptable or non-acceptable by the participants, and they also said they thought this could differ from workplace to workplace. The acceptable causes of sickness absence also differed from person to person, referring not only to the causes of sickness absence, but also the limits for calling in sick. Illnesses such as colds and stomach pain were to some extent acceptable reasons for sickness absence, but also disputable illnesses. An employee did not have to be absent because of it and some might disapprove of absence. Gastroenteritis and physical illnesses, i.e. like a broken arm and cancer, were agreed upon as acceptable illnesses for being off sick. Psychical illness like depression and stress-related disease were difficult to judge, and belonged to a grey zone of illnesses where it could be difficult to ‘see’ whether a person was ill or not.

The process of accepting a certain cause of sickness absence, where colleagues considered each other’s absences and the reasons for them, became a form of social and moral control. One participant described a situation where a colleague called in sick for an illness that someone else would have gone to work with. In the quote below, the participant suggests that some colleagues might call in sick too easily.

*But it varies greatly, you know, when people call in sick, and we are all different like that. Like, some may call in sick, and this isn’t something I know for a fact, but generally speaking, if they have a sore throat or something small like that, whereas others will come to work with a strep infection. Where do you draw the line? That’s a tough one, isn’t it…*

*Rural nursing home, group A, Denmark*

By categorizing illnesses, or sick colleagues, according to other colleagues’ perception and understanding of that illness, social limits and strains were produced. Work ethic and different causes of sickness absence were connected in some FGDs, and some causes of sickness absence seemed to be more easily accepted than others.

*You know, the work ethic is really very strong. Well, we don’t stay home unless it’s absolutely necessary. Like, when we’re throwing up or have a high fever, so the moral is that if you are really feeling bad, like when you’re sick, you get a doctor’s note.*

*Rural nursing home, group A, Norway*

Indisputable physical illnesses like strongly infectious diseases were easily and morally accepted reasons for sickness absence and notably also family problems such as divorce, children with problems or death of a close family member. The participants quoted here said explicitly that they had a high work ethic and were absent from work only when it was absolutely necessary. In both of the examples above, participants were talking about other colleagues who might choose to stay at home for a reason they themselves considered unacceptable for calling in sick. For example the degree of having flu or a cold was discussed. Some employees expressed that if they had flu and fever they would stay at home, if only flu they would go to work. This indicates a tendency towards inter-relational judgments and a form of social control.

Supporting a colleague in her sickness absence and/or sickness presenteeism was a form of positive social control. One participant described a situation where a colleague’s husband got cancer and how they supported her by facilitating her work.

*… Well, I remember with Martha, before, with her husband, how I thought about what it could be, but then she told us, that he had been diagnosed with cancer and all that, and then we carried her for quite a while, where we thought, though we never said anything to Martha, but that she shouldn’t have too many, or too much, because we could sense that she could just collapse completely if she got that, and then she’d be on sickness absence for real, right… .*

*Urban nursing home, group D, Denmark*

However, some conditions had to be met before the participants would consider supporting a sick colleague in her sickness absence or presenteeism. It required knowledge about why the colleague was not at work or why she could not perform all of her daily tasks if she was at work. The reason for her sickness absence or sickness presenteeism had to be accepted by her colleagues before they could support her. Colleagues could decide whether or not to accept a cause of sickness absence according to the severity of the illness, their perception of this severity, and their perception of the colleague herself. All these aspects were part of the same moral evaluation, and based on this, colleagues with sickness absence or sickness presence were assigned either a positive or negative moral position. The desire for social control, in that the sickness absence was acceptable in the eyes of colleagues, was evident in both countries. The participants expressed that acceptable reasons for sickness absence could, for some employees, differ due to life stages such as having children or not. It made an overall difference if the illness was visible or not. In one FGD they claimed it was easier to call in sick if you could see the illness, i.e. a broken arm, than if you had a psychological condition and did not look sick.

*Of course it’s easier to come in with a doctor’s note if you broke your arm, than if there’s something psychological and you can’t come to work. When nobody can see you’re sick. Of course it’s easier for people to stay home when they broke their arm. I think it’s a lot harder to come in with a sickness note when you look just fine…*

*Urban nursing home, group C, Norway*

The participants also said that even though they had access to technologies that assisted them in their work, many of them suffered from musculoskeletal disorders. Musculoskeletal disorders were experienced as something virtually inevitable, especially among older participants, and were considered acceptable causes of sickness absence and sickness presenteeism.

### Job identity

Job identity in this study was closely connected to relationships with colleagues and attitudes towards supervisors, but was especially connected to the employees’ commitment to their residents. Job identity emerged as a central topic when participants spoke about doing their job well, which basically implied giving good care plus a little extra to the residents. Indeed, the participants expressed a strong commitment to their residents, hoping to give them more than just basic care. Job identity was hence expressed in moral terms concerning how to perform well or bad in relation to significant others. One of the participants described that being able to do their work in a proper manner gave them a good job satisfaction:

*My goal for the day is to give the people I’m appointed to [*care for*] a good experience. And if I can do that, give them a good experience through a conversation, something we experience, good care, or whatever, I’ve had a good day. But if I have a very busy day, where I can’t give them those experiences, I deflate a little.*

*Rural nursing home, group A, Norway*

The participants expressed concerns about the residents and whether their residents’ needs would be met in a satisfactory manner if there were too few employees at work. This would end up affecting their job identity because the possibility to do their job properly would decrease if a colleague was sick. In most of the FGDs the participants expressed that they liked their work, and felt good about it if they were allowed to do it properly. This implied the presence of sufficient staff so they also had the time to talk with the residents, do the hair and nails of a female resident if she so wished, take them outside for a walk, etc. Younger and older participants in both countries made these statements in several FGDs both. To maintain and improve quality of life in addition to basic personal care, which every resident was entitled to, was cited as crucial in all FGDs. When partly sick colleagues or others, i.e. volunteers, came and did nice things in place of the employees, the job identity and work environment were affected in a negative way because employees who were not sick also wanted to do nice things, but felt they had no time.

The participants also expressed their worry for colleagues if there were too few employees at work.

*… Come on! We’re already only six tomorrow on weekend shift; if I’m sick, it will be below weekend staffing!*

*Rural nursing home, group A, Denmark*

Being too few for too long would wear and tear at the remaining staff, and negatively affect job identity. The participants also discussed the worsening of working conditions in recent years, partly because of tight budgets and health-wise poorer residents.

### Organization of work and physical aspects of the workplace

The organization of work, e.g. how work tasks were distributed among employees, was strongly affected by sickness absence and vice versa. This theme does not contain moral aspects in itself, but influenced and gave a context for how employees would reason morally about sickness absence and presenteeism. When a colleague called in sick or was at work partly sick, work tasks had to be rearranged in order to manage all the residents properly, or simply to attend to their basic needs:

*… but on an everyday basis, we almost know beforehand, if there’s people calling in sick, then you’ll just have to change the already planned workloads. And then we’ll have to work that much harder, and, depending on how many of us there are, we’ll talk about: "Will we have time to give baths today?"*

*Rural nursing home, group B, Denmark*

In both Norway and Denmark, it seemed quite common to rearrange the remaining employees when someone called in sick, because fewer employees were present. Sometimes it was not possible to summon a temporary employee, and then the remaining employees rearranged themselves in order to divide the work tasks in the best and most efficient way. Participants’ descriptions seemed to indicate that it was more difficult in Denmark than in Norway to summon a temporary employee because of budget strains. Participants from both Norway and Denmark reported that rearrangements happened almost daily.

Concerning the physical aspects of the workplace a few topics came up that seemed to be related to sickness absence and sickness presenteeism: the size of the nursing home, the age of the building, if the building had several floors, if lunch rooms were available and if the nursing home was located in a rural or urban area. One of the nursing homes was a small one in a rural area closely connected with the local community, where many residents of the nursing home were relatives or friends of people living in that community. The employees felt strongly committed to the local community and wanted to be looked upon both as a good place to live and to work. These close connections seemed to influence positively on their attitudes, and thus had an impact on the sickness absence. The employees knew each other well and the sickness absence rates were low. Nursing homes with different architecture added extra challenges when staff was reduced due to sickness absence. One of the nursing homes had several floors and the employees were supposed to be two in each floor, all together six employees. But when an employee reported sick there would often not be any extra help and they would be five to do the work of six. The different floors represented an additional challenge, causing the employees to become exhausted and distressed. We do not know if this directly contributed to more illness, but it affected our participants’ attitudes towards sickness absence and increased the sense of bustle and stress.

The four themes above are inter-related and constitute a different analytic scaling of the data. The first two themes take their starting point in being an employee and being sick, and focus on particular personal and moral aspects, behavioral patterns and reflections on causes for sickness absence and sickness presenteeism. The last two themes move away from these personal reflections on sickness *per se* and represent reflections on other factors related to sickness absence and presenteeism, such as job identity and the organization of work. Job identity especially seems to be related to the construction of moral attitudes and displays how specific attitudes overall are embedded in a common discourse on perception of job tasks, commitment and dedication to work. Organization of work is a contributory dimension to how to handle and perceive sickness absence which also influences specific moral positions.

## Discussion

We found that nursing home employees in Norway and Denmark readily discussed the implications of both sickness absence and sickness presenteeism, and that employees felt a strong commitment to both residents and colleagues. Relational dimensions were important when the participants discussed sickness absence and presenteeism, and a commitment to residents and colleagues came up as a natural part of it. The main points are that sickness absence and presenteeism were discussed and commented upon simultaneously by the participants as two sides of the same issue, that there is little chance of being spared heavy work tasks if one is partly sick, that it is difficult to have a sick colleague at work and no extra help due to heavy workloads, and that participants often had a bad conscience if they went to work partly sick, both towards their colleagues and because of their strong commitment to their residents. Social and moral control performed by colleagues and the nursing homes’ administrations, i.e., deciding what constituted acceptable and non-acceptable reasons for absence, was an important aspect of the relational dimension.

Managing sickness absence often included rearranging tasks among the remaining staff because extra help was seldom summoned. It was experienced as a larger problem to summon extra help in Danish nursing homes than in Norwegian nursing homes. The organization of the work was related to managing sickness absence through the rearranging of work tasks. Job identity emerged as a central theme when making decisions about sickness absence, and was heavily influenced by colleagues on sick leave. The management of sickness absence at the personnel level also influenced attitudes towards sickness absence and presenteeism. For example if it was possible to call for extra help, an employee could be off sick with a better conscience because she knew her absence would not make her colleagues work double. This seemed to affect individual attitudes towards sickness absence.

Sickness absence and presenteeism are complex issues with many layers of influence, e.g. structural factors connected to regulations and context [2, 33–35]. For example, job insecurity is more prevalent in Denmark compared to Norway and unemployment rates are higher in Denmark. Roelen and Groothoff [36] emphasized the importance of communication between supervisors and employees on sickness absence in order to facilitate their return to work. Finally, individual considerations concerning sickness absence and sickness presenteeism for one’s self and towards colleagues are important parts of these complex issues [37].

Our study results point more specifically to coherence between sickness absence and sickness presenteeism, in accordance with the findings of Elstad and Vabø [18]. Sickness absence and presenteeism are associated with each other, and one may lead to the other. Going to work when feeling ill was a significant risk factor for sickness absence [12] exceeding 30 days 3 years later [13]. In this study the choice to go to work sick and possibly be sent home, or to call in sick and stay at home was described as an unsolved dilemma.

The employees in our study felt a strong commitment to their residents and colleagues which is in agreement with the findings of Häggström et al. [38]. This strong commitment contributed to a bad conscience and poor job satisfaction when employees could not complete tasks as expected due to illness. But this same commitment led to a bad conscience regardless of whether employees went to work while partly sick or called in sick, and this was a difficult dilemma that they faced daily. A study of sick-listed employees showed that reporting sick is neither undertaken lightly, nor for short-term reasons only [39]. Our study supports this finding and the aspect of moral consideration was an important motivating factor in relation to the choices our participants made.

Sickness presenteeism is most prevalent in the health and care and education sectors, although sickness absence is also high in these occupations [7, 36]. A rigorous management of sickness absence will provoke sickness presenteeism, and sickness presenteeism is just as common as sickness absence [36]. The cost of sickness absence is significant in many countries [2], but the costs of productivity losses due to reduced work effort are higher than the costs of sickness absence [40]. In our study we found that sickness presenteeism was a very well-known option. Sickness presenteeism generally meant that the staff could not do their job as required, and it could be seen as a loss of productivity. Organizational cultures may differ and also influence attitudes toward sickness absence and sickness presenteeism, however our information was too limited to determine the validity of such a conclusion.

Another important aspect concerning sickness absence and sickness presenteeism is the perception of ill health [19]. The concept trilogy of illness, disease and sickness used by Wickman et al., is useful for capturing different aspects of ill health. Illness refers to how an individual identifies one’s own poor health, which is often based on self-perceived mental or physical symptoms. Disease, on the other hand, refers to a condition that is diagnosed by a physician or other medical expert. Sickness refers to another phenomenon, namely the social role a person with an illness or disease takes, or is given in society. Our study showed that the legitimate causes of sickness absence and the perceptions of ill health at the level of 'disease’ differed within the focus groups. One employee could express that it was acceptable to stay at home with sick children also when the entitled sick children’s days were used, while another found this non-acceptable. The reasons for differing attitudes seemed to be connected to different life stages, e.g. having children at different ages. Individual differences in the participants’ perceptions of when one was sick, and the limits according to when one should call in sick, were in general discussed among the employees on a daily basis.

However, the ability to work will, regardless of illness or disease, depend on the type of work and work demands [19]. Associations between sickness presenteeism and high workload, time pressure and job insecurity have been observed [7, 16, 41]. These factors are present in the health and care sectors, where both sickness absence and presenteeism are high. The importance of the relational dimensions of sickness absence we found in our study was also discussed in Wikman et al. [19]. One study found that health and care sector employees in Nordic countries reported worse physical and mental working conditions in 2005 compared to previous years [42]. This supports our findings; the participants in our study in both Norway and Denmark discussed that the working conditions had become worse in recent years, and there was for example less opportunity to summon temporary help. Physical surroundings, work climate and work pressure affected both the perceived level of sickness absence and sickness presenteeism, as shown in the theme Organization of work and physical aspects of the workplace.

When to call in sick depends on a combination of related pain, experience of health care, work- and labor-market-related factors and the relationship between self-image and work according to Hansson et al. [39]. The participants in our study discussed all these factors, and also job identity, which we see as similar to self-image at work. The employees expressed the importance of their close commitment to residents and that they experienced a satisfactory work day when they had the opportunity to perform their job properly. An important part of a good work day included time to take care of the residents, not only time to do the most urgent work tasks.

It is the illness or disease aspect of ill health that is referred to when an employee calls in sick. However, colleagues will ascribe the sick colleague a social role or, according to Dodier [5], a moral position which is used to develop social control. The employees figured out the moral position of their colleagues for example by discussing the reasons for sick leave with each other during work, and the relationships between the colleagues were important in the creation of a moral position. Sometimes an employee could express emotional resignation and signal that a colleague was on sick leave for the wrong reasons. This moral position might be understood as the link between the interaction and the social structure. Social interactions in the workplace take place every day, both before and after a doctor has labeled a person "sick" or "on sick leave", but the doctor permits her to stop working for a fixed period [5]. Calling in sick without going to the doctor does not carry optimal legitimacy with colleagues or the administration, and the moral evaluation of illness is different than for disease. The issue of legitimization of illness is closely related to feelings and this generates the problem of proving that one is ill [43]. The way an individual *feels* about his/her condition is understood as the prime criterion of health, illness and recovery. In our study we found that differences regarding when to call in sick for the same illness were discussed, i.e. attitudes to the notion of feeling sick for the same illness differed from person to person and from context to context. This was particularly relevant for common illnesses such as colds and flu.

Illnesses might be classified into different categories and a sick colleague will be put in one of these classifications depending on how the other colleagues perceive and understand the illness and the colleague. This produced social limits and strains, and the colleague was ascribed a social role.

In classifying illnesses into different categories as acceptable or non-acceptable reasons for sickness absence, the participants experienced and practiced social control. What constitutes a morally accepted reason for having sick leave might differ from person to person, as shown in our study. What is considered a morally acceptable reason for one colleague to stay at home is not necessarily accepted by the colleagues as a legitimate reason for another colleague; this is a manner of practicing social control. Colleagues as well as supervisors may practice this form of social control. The relational dimensions are important in practicing social control, and other aspects of the colleague’s life besides causes of sickness absence may be at stake in these situations. Social control is a dynamic concept that depends on individual and social factors. Unfortunately, this has been scarcely explored [5] and we have little knowledge about this important issue.

On the other hand, social relations and social emotions between absentees and family and work-life relations have proven to be important factors in absentees’ rehabilitation [44–46]. Knapstad et al. [44] found that increased understanding of the impact of social and emotional aspects around sickness absence could be an important source for improved quality of the return to work process.

### Strengths, limitations and future research

Attitudes towards sickness absence and sickness presenteeism in the health care sector are an unexplored field. Sickness behavior, reflecting attitudes on work absence, may differ between countries and influence rates. We have chosen to explore this in Norway and Denmark because the sickness absence rates are twice as high in Norway compared to Denmark. Furthermore, this study is part of a larger study in which the two countries are compared in relation to sickness absence levels, patterns and trends of absence [23, 26]. Because of the difference in sickness absence rates it was hypothesized that the attitudes towards sickness absence and presenteeism were also different in the two countries. However, the main strength of this study lies in its investigation of the field of sickness absence and sickness presenteeism within a reportedly vulnerable group in relation to sickness absence as a whole, which opens up for the significance of general moral dilemmas. FGDs were carried out at different nursing homes to ensure diversity in the responses, but the analysis did not aim for a comparison of specific parameters. Obviously, one focus group with only two participants turned into a group interview rather than a discussion between the participants. There is a balance between what constitutes a FGD and a group interview which is relevant to data generation [28].

To interpret the data as accurately as possible, interviews were done and interpreted by researchers from Norway and Denmark. Consistency of results and credibility/trustworthiness are important validity aspects of interview studies. The three different positions of the authors who took part in the analysis workshop was an attempt to triangulate between researchers, e.g. finding consistency between data and findings and discussing deviant cases. Findings were not presented to the participants of the study. Disagreements on findings were resolved during workshop or in consultation with author RJ. Prior to the workshop, the whole study setting, aim and design had been discussed and developed in an interdisciplinary team consisting of epidemiologists, health service researchers and sociologist/anthropologists. Also throughout the interview process, answers and findings were continually scrutinized and reflected by the main researchers to control for validity. Finally, the findings were validated in terms of communicative validity reflecting other findings, theory and positions in the research community [47]. Both internal and external validity in qualitative research is a continuous process which we have attempted to engage in and it is not merely a final product validation. In the end, interpretations rely on the strength of the argument that is offered for discussion.

There are limitations to the current study, including those in the interpretation of the results of the study. The focus groups turned out to be different than expected, especially with regard to age. The divide between young and older employees was difficult to implement in practice in the nursing homes. In one of the nursing homes there were no employees less than 40 years old, but as they had been working in nursing homes for many years, they did not conceive themselves as young. The employees themselves thought of employees around 20 years of age as "young". This was important knowledge that came from the FDGs. It could have been useful to interview organizational leaders to study if their attitudes towards sickness absence and sickness presenteeism differed from the nursing assistants, but this is for further studies.

Who selected the participants is important and, in this case it was the administrations of the nursing homes themselves. In addition, it was difficult for some administrations to find participants among the youngest employees, i.e. in their 20s.

Focus groups tend to give information about what is acceptable to say. We do not know if this is in compliance with what is practiced or said in other work-related contexts. Thus, we have to interpret the data in a trustworthy and transparent way as we have done in this study by using several researchers and workshop in the interviews and interpretation, and by comparing the interpretation of the data with relevant literature.

This study highlights the importance of awareness of attitudes towards sickness behavior both as premises for the sickness level within working places, sectors, countries, and between genders and age groups, and for understanding effects of changing regulations and legislations. Nursing home assistants do not have the type of job where work can be taken home or made up later, so the consequences of the attendance decisions are immediate, as illustrated in the results. This study cannot shed light on sickness behaviour associated with jobs where work can be taken home or made up at a later time.

This study may lead to more in-depth fieldwork on the subject, i.e. participant observation and one-to-one interviews. Future research is needed to study when employees choose sickness absence over sickness presenteeism, or vice-versa. Other aspects that need to be studied are how social control is practiced in the workplace through attitudes towards sickness absence and sickness presenteeism in general, and towards sick employees in particular; how supervisors relate to sickness absence and sickness presenteeism and whether this is different in Norway compared to Denmark. More knowledge about the relational dimensions will bring us a step forward in understanding the phenomenon of sickness absence and sickness presenteeism.

## Conclusions

The general interpretation of the findings was that attitudes towards sickness absence and sickness presenteeism among nursing home employees were embedded in situational patterns of moral relationships and were connected to a specific job identity. These patterns were constituted by the perception of colleagues, the social commitment to residents, and they influence on what was deemed as acceptable and non-acceptable reasons for sickness absence. In other words, attitudes to sickness absence and sickness presenteeism were socially and morally determined at personal levels by an overall concept of work, independent of country.

## Abbreviations

FGD: Focus group discussion.
